# Evaluation of Extraction and Degradation Methods to Obtain Chickpeasaponin B1 from Chickpea (*Cicer arietinum* L.)

**DOI:** 10.3390/molecules22020332

**Published:** 2017-02-21

**Authors:** Kun Cheng, Hua Gao, Rong-Rong Wang, Yang Liu, Yu-Xue Hou, Xiao-Hong Liu, Kun Liu, Wei Wang

**Affiliations:** School of Pharmacy, Qingdao University, Qingdao 266021, Shandong, China; chengkun1990518@163.com (K.C.); gaohuaqy@126.com (H.G.); rrwang2012@163.com (R.-R.W.); buckuper@163.com (Y.L.); hyx19931008@163.com (Y.-X.H.); liuxiaohong1043@163.com (X.-H.L.); kunliu62@126.com (K.L.)

**Keywords:** *Cicer arietinum* L., chickpea, chickpeasaponin B1, microwave-assisted extraction, alkaline hydrolysis

## Abstract

The objective of this research is to implement extraction and degradation methods for the obtainment of 3-*O*-[α-l-rhamnopyranosyl-(1→2)-β-d-galactopyranosyl] soyasapogenol B (chickpeasaponin B1) from chickpea. The effects of microwave-assisted extraction (MAE) processing parameters—such as ethanol concentration, solvent/solid ratio, extraction temperature, microwave irradiation power, and irradiation time—were evaluated. Using 1g of material with 8 mL of 70% aqueous ethanol and an extraction time of 10 min at 70 °C under irradiation power 400W provided optimal extraction conditions. Compared with the conventional extraction techniques, including heat reflux extraction (HRE), Soxhlet extraction (SE), and ultrasonic extraction (UE), MAE produced higher extraction efficiency under a lower extraction time. DDMP (2,3-dihydro-2,5-dihydroxy-6-methyl-4*H*-pyran-4-one) saponin can be degraded to structurally stable saponin B by the loss of its DDMP group. The influence of pH and the concentration of potassium hydroxide on transformation efficiency of the target compound was investigated. A solution of 0.25 M potassium hydroxide in 75% aqueous ethanol was suitable for converting the corresponding DDMP saponins of chickpeasaponin B1. The implementation by the combining MAE technique and alkaline hydrolysis method for preparing chickpeasaponin B1 provides a convenient technology for future applications.

## 1. Introduction

**Figure 1:**
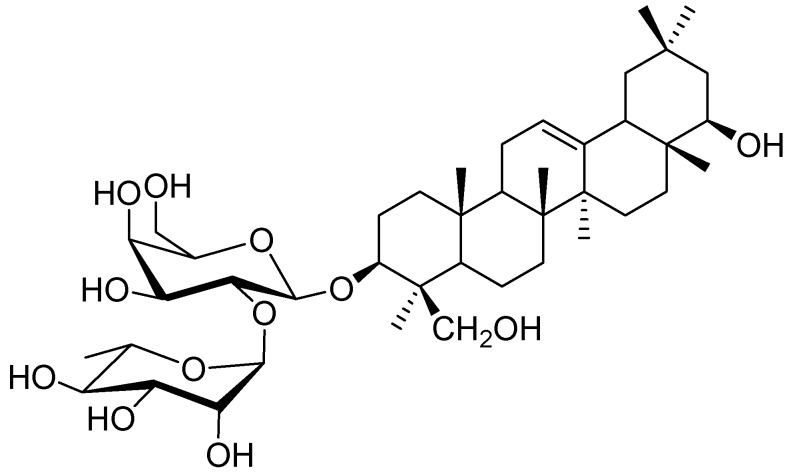
Structure of chickpeasaponin B1.

Saponins, isolated from legumes, consist of a complex mixture of pentacyclic triterpenoid glycosides. For example, soybeans contain two different types of genuine saponins that are classified into group A saponins and 2,3-dihydro-2,5-dihydroxy-6-methyl-4*H*-pyran-4-one (DDMP) saponins [1,2]. Group A saponins that have soyasapogenol A (3β,21β,22β,24-tetrahydroxyolean-12-ene) as an aglycone are bisdesmosides, while DDMP saponins that have soyasapogenol B (3β,22β,24-trihydroxyolean-12-ene) as an aglycone are monodesmosides [3,4,5,6,7,8,9,10,11]. DDMP saponins degrade into non-DDMP conjugated group B and E saponins, which is dependent on solvent, temperature, and storage time [12,13,14]. Group B saponins are the major saponins in processed soybean products, due to this conversion during extraction and processing [14]. Although soybean saponins have been analyzed, studies are ongoing to elucidate the chemical structures and biological activities of saponins in several other species of the legume family [15,16,17,18,19,20]. Chickpeas (*Cicer arietinum* L.), one of the wild progenitors of seven Neolithic founder crops and the third largest legume with respect to planting area for human consumption, are common in the Mediterranean region, the Middle East, South and Central America, India, Pakistan, Bangladesh, and China [21,22,23]. In addition to culinary usage, chickpeas have been accepted as a natural Uighur traditional medicine in Xinjiang (China) for over 2500 years [24]. Literature data reported that chickpeas contain mainly soyasaponin βg, a DDMP saponin, that was identified by fast atom bombardment mass-spectrometry [25]. A microwave-assisted extraction (MAE) method was developed for the extraction of soyasaponin βg from chickpea [26]. In the course of our study, the chickpeas purchased from an agricultural products market in Urumqi, China, were extracted using the MAE method reported by Kerem, et al. (2005). However, we found the retention time of the major component in the chickpea extract is different from that of soyasaponin βg standard by HPLC analysis. So, we also carried out a chemical investigation on *C*. *arietinum*, which resulted in the isolation of a new saponin, chickpeasaponin B1 (Figure 1). This was found to be a major component of the chickpea extract. The structure was determined as 3-*O*-[α-l-rhamnopyranosyl-(1→2)-β-d-galactopyranosyl] soyasapogenol B on the basis of spectroscopic analysis as well as high resolution mass spectrometry and acid hydrolysis (NMR spectral data of chickpeasaponin B1 are available in the supplementary materials online). It was found to have a unique feature in legumes, that is, the presence of the 1→2 interglycosidic linkage between rhamnose and galactose. Chickpeasaponin B1 may be an artifact degraded from the corresponding DDMP saponins during the extraction and isolation procedures. Because of the structural stability of chickpeasaponin B1, we expect it is the source of chickpea functionality rather than DDMP saponin. Furthermore, the beneficial effects such as hypoglycemic, hepato-protective, anti-inflammatory, and anti-carcinogenic activities have been revealed using group B soyasaponins [27,28,29,30].

Saponins extracted from chickpeas could add value to the chickpea processing industry. The first step of processing is extraction, which involves separating the saponins from the cellular matrix of the chickpea. As an extraction technique, MAE utilizes the energy of microwaves to cause movement of molecules with a permanent dipole. This causes the internal temperature of each cell to rise rapidly and the cell to rupture due to the increased internal pressure. Thus, the extraction components are freely discharged. This process offers many advantages such as less solvent consumption, less extraction time, high extraction rate, and strong applicability to industrial settings [31,32]. In recent years, MAE technique has become very popular for extraction of bioactive constituents from natural products [33,34,35,36,37,38,39,40,41,42]. In this study, the objectives are to employ MAE to extract all the saponins in chickpeas and to compare rapid degradation conditions that convert corresponding DDMP saponins into chickpeasaponin B1. The extraction efficiency of MAE was compared with three other conventional extraction processes. The morphology of chickpea powder processed by MAE was observed using scanning electron microscopy. The effects of extraction and degradation processing parameters—such as ethanol concentration, solid/liquid ratio, extraction temperature, microwave irradiation power, irradiation time, pH, and concentration of potassium hydroxide—were evaluated as well. The chickpeasaponin B1 was quantified by HPLC to evaluate extraction and degradation yields.

## 2. Results and Discussion

### 2.1. Optimization of MAE Conditions

The univariate method was used to optimize the parameters of MAE including ethanol concentration, solid/liquid ratio, extraction temperature, microwave irradiation power, and irradiation time, any of which could extensively affect the extraction efficiency. 

#### 2.1.1. Effect of Ethanol Concentration

**Figure 2:**
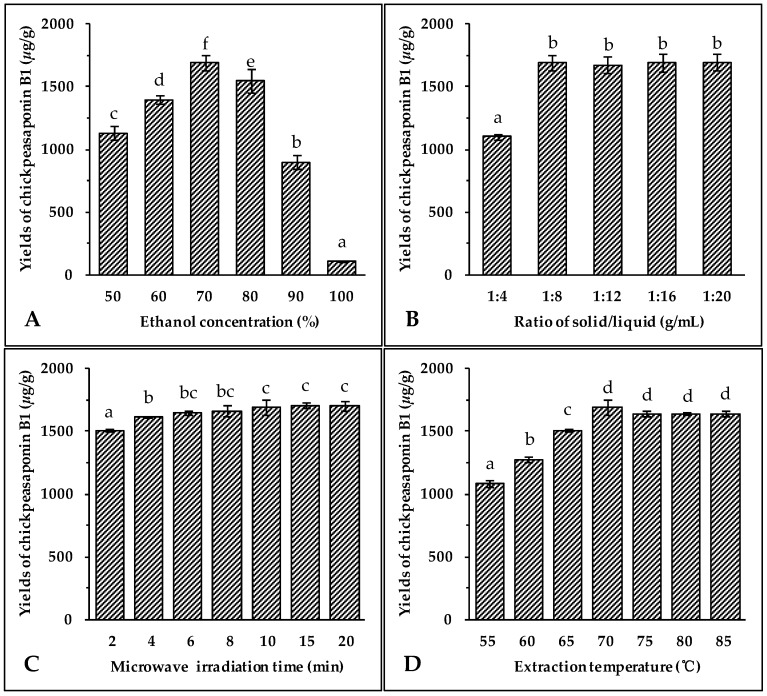

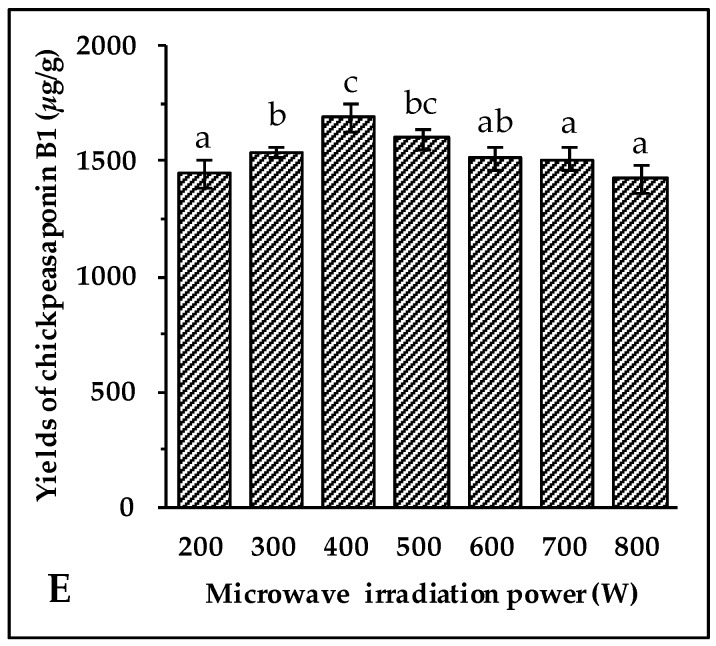
Effect of ethanol concentration (**A**); solid/liquid ratio (**B**); irradiation time (**C**); extraction temperature (**D**); and microwave irradiation power (**E**) on the yields of target compound. Data are expressed as the mean value ± standard deviation (S.D.) of triplicate experiments. Data marked with different letters “a”, “b”, “c”, “d”, “e”, or “f” have significant differences, *p* < 0.05, while those marked with the same letter denote no significant differences, *p* > 0.05. Data marked with “ab” or “bc” have no significant differences from those marked with “a” or “b”, “b” or “c”, *p* > 0.05, respectively.

The selection of the most appropriate solvent for extracting the target compounds from the sample matrix is an essential step for developing any new extraction method. As a non-toxic and pollution-free choice, ethanol is regularly used for the extraction of natural products [43,44,45]. It has been observed that the addition of small amounts of water to the extraction solvent often helps to increase the extraction yield of the target compounds from the sample [44,45,46]. Therefore, aqueous ethanol was chosen as the extraction solvent. Different concentrations of ethanol (50%, 60%, 70%, 80%, 90% and 100%) were used as solvents to extract total saponins from chickpea. Other extraction conditions were held constant: the solid to liquid ratio of 1:8 (g/mL), microwave irradiation power of 400 W, extraction temperature of 70 °C, and extraction time of 10 min. The results illustrated in Figure 2A showed that the highest extraction yield of chickpeasaponin B1 was obtained at 70% aqueous ethanol. When the concentration of ethanol was between 50% and 70%, the yield increased with the increasing concentration of ethanol. The yield declined when using 70% to 100% of ethanol. Thus, 70% aqueous ethanol was selected as the optimal solvent for the following extraction experiments.

#### 2.1.2. Effect of Solvent/Solid Ratio

Large ratios of extraction solvent to substrate would lead to unnecessary waste, while small ratios may lead to incomplete extraction. Thus, the influence of the ratio of solvent to material on extraction efficiency of the target compound was evaluated next. The solvent used was 70% aqueous ethanol, microwave irradiation power was 400 W, extraction temperature was 70 °C, and time of extraction was 10 min. Data shown in Figure 2B indicated an obvious increase of extraction yield of chickpeasaponin B1 when the solvent to solid ratio was increased from 4 to 8 mL/g. When the solvent to solid ratio was increased from 8 to 20 mL/g, however, no significant difference in the extraction yield of chickpeasaponin B1 was detected. For commercial application, a solvent to solid ratio of 8 mL/g should be optimal for avoiding waste of solvent and excess work in the concentration process.

#### 2.1.3. Effect of Irradiation Time

Microwave irradiation is able to generate sufficient energy in a short time, leading to an equilibrium between the objective constituents inside and outside the plant cell. The effect of the extraction time on chickpeasaponin B1 yield was investigated, and other experimental parameters were set as follows: ethanol concentration, 70%; liquid to solid ratio, 8:1 (mL/g); microwave irradiation power, 400 W; and extraction temperature, 70 °C. The results shown in Figure 2C clearly indicated that the extraction yield of chickpeasaponin B1 increased from 1505.31 ± 10.22 μg/g to 1692.27 ± 63.72 μg/g when extraction time increased from 2 to 10 min. However, the difference in the yield of chickpeasaponin B1 was not significant (*p* > 0.05) when the time of MAE increased from 10 to 20 min. Because the diffusion front moved towards the interior of the tissues, the diffusion area reduced, diffusion distance increased, and the diffusion rate decreased accordingly [47]. Therefore, there was no obviously-observed yield change during the prolonged time period and 10 min was chosen as optimal extraction time.

#### 2.1.4. Effect of Extraction Temperature

Extraction temperature impacts the solubility and mass transfer rate of the target compound. Seven different temperatures (55, 60, 65, 70, 75, 80 and 85 °C) were selected to evaluate the influence of the temperature on the extraction efficiency of chickpeasaponin B1 from chickpea. Seven groups of samples were pretreated with the optimal parameters obtained in the previous assays: 70% aqueous ethanol, 10 min of extracting time, and an 8:1 ratio of solvent to solid. As the temperature increased from 55 °C to 70 °C, the yields of chickpeasaponin B1 significantly increased (Figure 2D). However, when the temperature exceeded 70 °C, the yields of chickpeasaponin B1 decreased. This extreme temperature might have caused the chickpea powder with high starch content to partially cook, thereby causing the loss of chickpeasaponin B1 [44]. So, 70 °C was found to be the extraction temperature that produced the highest yield.

#### 2.1.5. Effect of Irradiation Power

Microwave energy affects the molecular interactions between the solvent and the objective compounds significantly. Under the above optimal conditions of extraction, 70% aqueous ethanol solvent, 10 min of irradiation, an 8:1 ratio of liquid to material, and an extraction temperature of 70 °C, the experiment was carried out at 200, 300, 400, 500, 600, 700, and 800 W, respectively. As shown in Figure 2E, when the irradiation power increased from 200 W to 400 W, the extraction yields of chickpeasaponin B1 increased significantly, while the yields showed the opposite trend when the power increased from 400 W to 800 W. It is speculated that the superfluous energy offered by high irradiation power disturbed the molecular interaction, which was adversarial to the extraction of chickpeasaponin B1. Therefore, it was appropriate to select 400 W as the practical microwave irradiation power with the yield of 1692.27 ± 63.72 μg/g.

### 2.2. Comparison of Different Extraction Methods

In order to evaluate the extraction efficiency of MAE, the extraction yield was compared with conventional extraction methods like heat reflux extraction (HRE), Soxhlet extraction (SE), and ultrasonic extraction (UE). The extraction results of chickpeasaponin B1 with varying times and extraction methods are shown in Table 1. The results showed that, compared with HRE at 120 min, SE at 210 min, and UE at 90 min, the highest extraction yield of chickpeasaponin B1 was achieved by MAE at 20 min. MAE yielded chickpeasaponin B1 (1692.27 ± 63.72 μg/g) only when the extraction time reached 10 min. For HRE, when the extraction time exceeded 60 min, the yield of chickpeasaponin B1 tended to remain constant. Times of 210 min and 90 min were needed for SE and UE to produce their maximum extraction yield of 1079.11 ± 59.50 μg/g and 871.28 ± 27.92 μg/g, respectively, and these were lower than that of MAE (*p* < 0.01). MAE took less time and had a higher extraction yield than other methods. Therefore, MAE was found to be suitable for the extraction of total saponins from chickpea.

### 2.3. Scanning Electron Microscopy Observation

**Figure 3:**
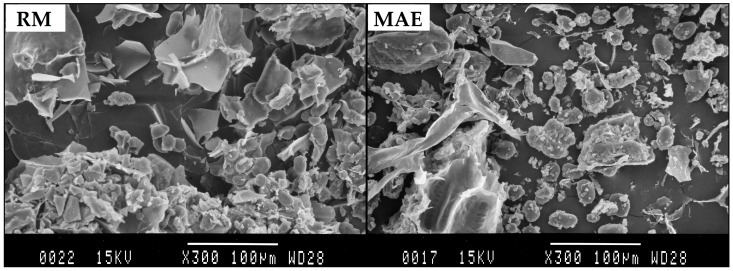
Left, a scanning electron microscopy image of a sample of raw material (RM) and right, the sample processed by MAE.

In order to evaluate the morphological alteration after the MAE process, the samples were observed with SEM. Figure 3 shows micrographs of a sample of raw material (RM) and a sample after MAE for 10 min. The micrograph of the RM revealed numerous spherical or elliptical particles. Compared with RM, chickpea powder after MAE treatment contained the amorphous material, spherical particles, and clear fragmentation of cell walls. This observation confirmed that, during microwave irradiation, rapid heating of polar molecules in the material and the solvent led to a rapid expansion of the solvent volume, which increased the pressure within the cells and caused an explosive release of the substances inside of the plant cells [48].

### 2.4. Comparison of Degradation Methods

**Figure 4:**
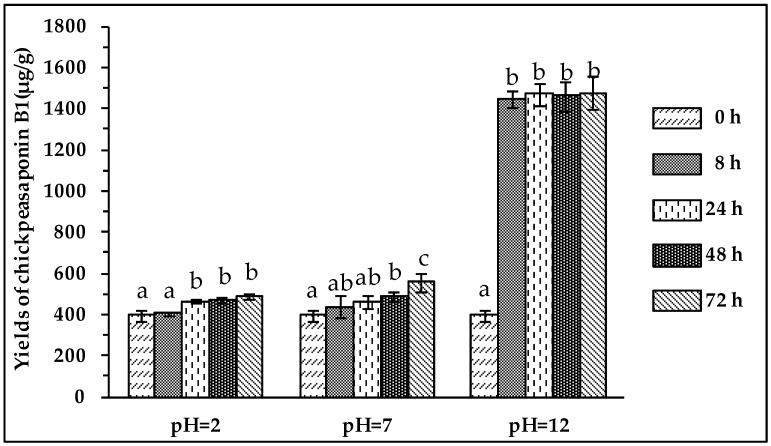
Effect of pH on the yields of target compound. Data are expressed as the mean value ± standard deviation (S.D.) of triplicate experiments. Data marked with different letters “a”, “b”, or “c” have significant differences, *p* < 0.05, while those marked with the same letter denote no significant difference, *p* > 0.05. Data marked with “ab” have no significant differences with those marked with “a” or “b”, *p* > 0.05, respectively.

**Figure 5:**
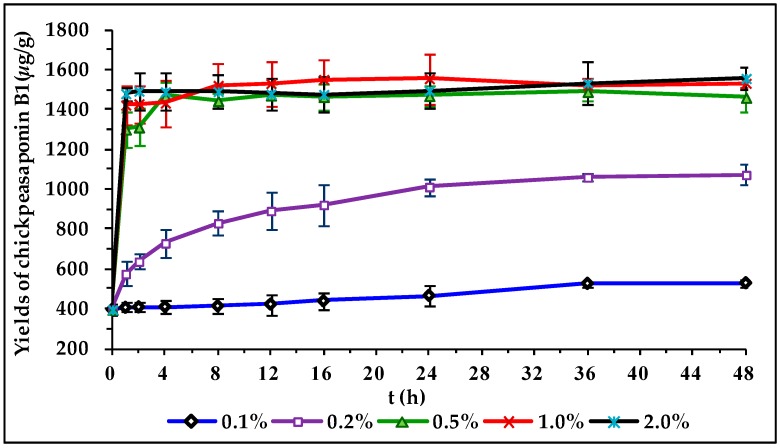
Hydrolysis dynamic curves of the total saponins of chickpea catalyzed by potassium hydroxide in 75% aqueous ethanol with different concentrations of 0.1%, 0.2%, 0.5%, 1.0%, 2.0%. Data are expressed as the mean value ± standard deviation (S.D.) of triplicate experiments.

**Figure 6:**
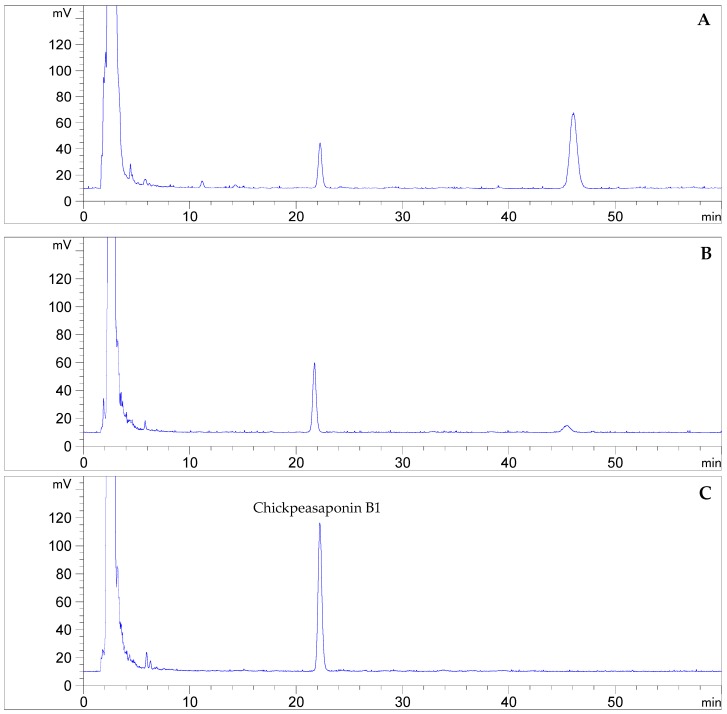
Quantification by HPLC-ELSD of chickpeasaponin B1 obtained from chickpea extracted by MAE (**A**); the total saponins degraded by potassium hydroxide in 75% aqueous ethanol (1%, g/mL) for 0.5 h (**B**); and 12 h (**C**).

To degrade the corresponding DDMP saponin to chickpeasaponin B1, the removal of the DDMP moiety from the structure had to be targeted. The effect of pH on the stability of DDMP saponin was studied in this experiment. As shown in Figure 4, it was found that alkaline hydrolysis in aqueous ethanol produced the maximum amount of chickpeasaponin B1 when compared to aqueous ethanol and acid hydrolysis in aqueous ethanol. This result was partly consistent with the findings reported by Zhang et al. (2009), who transformed the DDMP-conjugated group B soyasaponins into the non-DDMP-conjugated soyasaponins I and III [12]. The amount of chickpeasaponin B1 produced by basic 75% aqueous ethanol (pH = 12) increased the level of chickpeasaponin B1 by approximately 1079.67 μg/g chickpea or an increased percentage of 272.74%. To select a proper concentration of potassium hydroxide in 75% aqueous ethanol that would guarantee completion degradation of corresponding DDMP saponins of chickpeasaponin B1, hydrolysis dynamics curves were investigated (Figure 5). It was observed that when the potassium hydroxide concentrations were 0.1% and 0.2%, the converting rates of the corresponding DDMP saponins reached their maximum with the yields of chickpeasaponin B1 of 530.14 ± 18.37 μg/g and 1072.72 ± 51.34 μg/g at 48 h, were produced respectively. When the concentration was increased to 0.5%, catalytic efficiency was significantly enhanced, and most of the corresponding DDMP saponins of chickpeasaponin B1 were transformed in 12 h. The comparison between the first and the last chromatogram in Figure 6 indicated that the corresponding DDMP saponins had been hydrolyzed completely to chickpeasaponin B1.

## 3. Materials and Methods

### 3.1. Materials and Reagents

Chickpeas were purchased from an agricultural products market in Urumqi, China, and authenticated by Prof. Ying-Xia Li, Qingdao University. A voucher specimen was deposited in Natural Products Research Laboratory, School of Pharmacy, Qingdao University. Chickpeas were ground into a powder using an electric disintegrator (FW177, Tianjin Taisite Instruments Co., Ltd., Tianjin, China). The ground powder was pass through a stainless steel sieve for homogenization (20 mesh) and stored in airtight bags at 4 °C for later use. Reference compound chickpeasaponin B1 with more than 98% purity was isolated and purified from the seeds of *C. arietinum* in our laboratory. The structure was determined as 3-*O*-[α-l-rhamnopyranosyl-(1→2)-β-d-galactopyranosyl] soyasapogenol B on the basis of spectroscopic analysis as well as high resolution mass spectrometry and acid hydrolysis. Acetonitrile and methanol of chromatographic grade were bought from Tedia Company Inc. (Fairfield, OH, USA). Deionized water was purified by a Milli-Q water purification system from Millopore (Bedford, MA, USA). All reagents and samples prepared for chromatographic analysis were filtered through 0.45 μm membranes (Jinteng Experimental Equipment Co., Ltd., Tianjin, China) before use. All other reagents obtained from Fuyu Fine Chemical Co., Ltd. (Tianjin, China) were of analytical grade.

### 3.2. HPLC Analysis

HPLC analyses were performed on an Agilent 1260 Infinity HPLC system equipped with a G1311C quaternary pump, a G1329B autosampler, a G1316A thermostatted column compartment, and a G4260B evaporative light scattering detector (ELSD) coupled with an analytical workstation (Agilent Technologies, Inc., Santa Clara, CA, USA). Chromatographic separation was achieved on a YMC-Pack ODS column (250 mm × 4.6 mm i.d., 5 μm, YMC Co., Ltd., Kyoto, Japan). Methanol–acetonitrile–water containing 2% (*v*/*v*) acetic acid (40:25:35, *v*/*v*/*v*) was used as the mobile phase. The flow rate was 1.0 mL·min^−1^, the injection volume was 5 μL, and the column temperature was maintained at 25 °C. For ELSD detection, the carrier gas was air, the evaporating tube and atomization tube temperatures were set at 110 °C and 80 °C, respectively, with gas flow rate of 1.4 L·min^−1^. The chromatographic peak of chickpeasaponin B1 was verified by comparing its retention time with the reference standard. The content was calculated from the working calibration curve constructed by plotting the logarithm of peak areas vs the logarithm of the amounts injected series reference standard solutions.

### 3.3. Comparison of Extraction Methods

#### 3.3.1. Microwave-Assisted Extraction (MAE)

MAE was carried out in a MAS-II microwave auxiliary reaction/extraction system (Shanghai Sineo Microwave Chemistry Technology Co., Ltd., Shanghai, China). An accurately weighed 10 g sample of chickpea powder was mixed with 80 mL of 70% aqueous ethanol in a special microwave three-neck flask (300 mL), which was put in the microwave-assisted extraction apparatus and connected to a condensing unit and temperature control device for microwave-assisted extraction. The extractions were conducted at a temperature of 70 °C with a microwave irradiation power of 400 W for 2, 4, 6, 8, 10, 15, and 20 min.

#### 3.3.2. Heat Reflux Extraction (HRE)

An accurately weighed 10 g sample of chickpea powder was moved into a round-bottomed flask equipped with a water condenser tube. The 70% aqueous ethanol was used as the extraction solvent, and the ratio of solvent to material was 8:1. The reflux extractions were carried out at a temperature of 90 °C in a thermostat boiling-water bath, and with the time periods of 10, 20, 30, 60, 90, 120, and 150 min.

#### 3.3.3. Soxhlet Extraction (SE)

An accurately weighed 10 g sample of chickpea powder was packed into a cardboard tube to a suitable height using filter paper. It was then placed into a Soxhlet extraction apparatus. The Soxhlet extraction apparatus was connect to a reflux condenser, fixed in a thermostat boiling-water bath followed by the addition of 80 mL of 70% aqueous ethanol. The experimental temperature was set at 90 °C for 30, 60, 90, 120, 150, 180, and 210 min.

#### 3.3.4. Ultrasonic Extraction (UE)

To study extraction by ultrasonication, an accurately weighed 10 g sample of chickpea powder was transferred in a flat-bottomed flask containing 70% aqueous ethanol (80 mL). The flask was immersed in an ultrasonicator bath (KQ-500E, Kunshan Ultrasonic Instruments Co., Ltd., Kunshan, China, 40 kHz, 500 W) and ultrasonicated for 10, 20, 30, 40, 50, 60, 90, and 120 min.

### 3.4. Optimization of MAE Conditions

In order to determine the best extracting medium for saponins in chickpea, a 10 g sample was mixed with aqueous ethanol in concentrations of 50%, 60%, 70%, 80%, 90%, and 100%, respectively. The best extracting solvent was examined and the ratios of solvent to material with the proportions of 4, 8, 12, 16, and 20 (*v*/*w*, mL/g) were tested. Optimization of irradiation time was performed with the designed periods of 2, 4, 6, 8, 10, and 12 min. To study the effects of extraction temperature and microwave irradiation power, 10 g of powder was subjected to 80 mL of 70% aqueous ethanol and irradiated at different temperatures including 55, 60, 65, 70, 75, 80, and 85 °C with microwave irradiation power of 400 W. Different power levels of 200, 300, 400, 500, 600, 700, and 800 W at the extraction temperature of 70 °C were also tested.

### 3.5. Extract Concentration

Once the extraction processes were accomplished, the extracted solutions were filtered and 50 mL of this solution were concentrated using a rotary evaporator (N-1001, Tokyo Rikakikai Co., Ltd., Tokyo, Japan) and stored at −20 °C for further testing.

### 3.6. Comparison of Degradation Methods

The solvent-free extract was dissolved and diluted with acidic 75% aqueous ethanol (pH = 2) neutral 75% aqueous ethanol (pH = 7), and basic 75% aqueous ethanol (pH = 12) in a 10 mL volumetric flask, respectively. The mixture was hydrolyzed for the time periods of 8, 24, 48, and 72 h at room temperature. To investigate the effect of the concentration of potassium hydroxide in 75% aqueous ethanol, the sample was subjected to 10 mL of various concentrations of sodium hydroxide (0.1%, 0.2%, 0.5%, 1.0%, 2.0%) and incubated for 1, 2, 4, 8, 12, 16, 24, 36, and 48 h at room temperature.

### 3.7. Statistical Analysis

Data were expressed as the mean ± standard deviation (S.D.) of triplicate determination. Statistical calculations were analyzed by one-way analysis of variance using the SPSS version 13.0 software (SPSS, Inc., Chicago, IL, USA). A value of *p* < 0.05 was considered statistically significant.

## 4. Conclusions

In summary, an efficient process for obtainment of chickpeasaponin B1 from chickpeas was established using the combining MAE technique and alkaline hydrolysis method. The procedures were optimized, validated, and compared with other conventional processes. High quality product containing mainly chickpeasaponin B1 was produced with less production time duration. Therefore, the results indicated the feasibility for future applications.

## Figures and Tables

**Table 1:** Extraction efficiency of four processes on chickpeasaponin B1, including microwave-assisted extraction (MAE), heat reflux extraction (HRE), Soxhlet extraction (SE), and ultrasonic extraction (UE).

MAE		HRE	
Extraction Time (min)	Yields (μg/g)	Extraction Time (min)	Yields (μg/g)
2	1505.31 ± 10.22	10	1277.34 ± 19.39
4	1613.20 ± 7.80	20	1433.34 ± 17.94
6	1651.91 ± 17.64	30	1490.64 ± 70.29
8	1666.89 ± 43.85	60	1557.67 ± 54.23
10	1692.27 ± 63.72	90	1610.18 ± 23.13
15	1710.83 ± 20.88	120	1628.20 ± 33.29
20	1703.22 ± 35.60	150	1595.12 ± 35.37
**SE**		**UE**	
**Extraction Time (min)**	**Yields (** **μ** **g/g)**	**Extraction Time (min)**	**Yields (** **μ** **g/g)**
30	212.28 ± 10.96	10	434.41 ± 46.11
60	246.80 ± 42.79	20	479.95 ± 20.65
90	373.81 ± 52.17	30	675.25 ± 26.80
120	493.67 ± 40.35	40	690.49 ± 29.26
150	599.63 ± 44.92	50	708.59 ± 35.87
180	776.00 ± 67.17	60	773.49 ± 22.92
210	1079.11 ± 59.50	90	871.28 ± 27.92

Data are expressed as the mean value (± SD) of three independent experiments.

## Supplementary Materials

Supplementary materials are available online.
