# Supplementary material for: Evaluation of Extraction and Degradation Methods to Obtain Chickpeasaponin B1 from Chickpea (Cicer arietinum L.)
Source: Molecules. 2017 Feb 21;22(2):332. doi: 10.3390/molecules22020332 (PMC6155701; doi:10.3390/molecules22020332)
Supplement: Supplementary file 1 [file molecules-22-00332-s001.pdf]

Supplementary Material: Evaluation of Extraction and Degradation Methods to Obtain Chickpeasaponin B1 from Chickpea (*Cicer arietinum* L.)

Kun Cheng, Hua Gao, Rong-Rong Wang, Yang Liu, Yu-Xue Hou, Xiao-Hong Liu, Kun Liu, and Wei Wang

Supporting information

Table S-1. ^{1}H NMR Data of the aglycone and monosaccharide moiety of chichpeasaponin B1.
Table S-2. ^{13}C NMR Data of the aglycone and monosaccharide moiety of chichpeasaponin B1.

Table S-1. ^{1}H NMR Data of the aglycone and monosaccharide moiety of chichpeasaponin B1 in DMSO-d_6.

Aglycone		Monosaccharide	
1	0.90 and 1.51, 2H[b]	1'	4.74, 1H, d (7.0)
2	1.70 and 1.79, 2H[b]	2'	3.41, 1H[b]
3	3.23, 1H[b]	3'	3.50, 1H[b]
4		4'	3.54, 1H[b]
5	0.84, 1H[b]	5'	3.35, 1H[b]
6	1.28 and 1.51, 2H[b]	6'a	3.47, 1H[b]
7	1.30 and 1.45, 2H[b]	6'b	3.51, 1H[b]
8		1''	4.95, 1H, br s
9	1.47, 1H[b]	2''	3.65, 1H[b]
10		3''	3.53, 1H[b]
11	1.78 and 1.79, 2H[b]	4''	3.14, 1H[b]
12	5.16, 1H, br s	5''	3.89, 1H[b]
13		6''	1.09, 3H, d (6.0)
14			
15	0.93 and 1.67, 2H[b]		
16	1.13 and 1.65, 2H[b]		
17			
18	1.99, 1H, br d (13.2)		
19	0.88 and 1.67, 2H[b]		
20			
21	1.28 and 1.30, 2H[b]		
22	3.23, 1H[b]		
23	1.13, 3H, s		
24	3.07 and 3.91, 2H, d (10.8)		
25	0.80, 3H, s		
26	0.88, 3H, s		
27	1.05, 3H,s		
28	0.75, 3H, s		
29	0.84, 3H, s		
30	0.97, 3H, s		

[a]Assignments were established by HSQC spectrum. *J* values (in hertz) are given in parentheses. [b]Multiplicities not assigned due to overlapped signal.

Table S-2. ^{13}C NMR Data of the aglycone and monosaccharide moiety of chichpeasaponin B1 in DMSO-d_6.

Aglycone		Monosaccharide	
1	38.0	1'	100.0
2	25.6	2'	75.6
3	90.0	3'	70.6
4	43.0	4'	69.2
5	55.1	5'	74.5
6	17.8	6	59.8
7	32.6	1''	100.2
8	40.0	2''	70.6
9	46.8	3''	74.6
10	35.8	4''	72.4
11	23.1	5''	67.9
12	121.5	6''	17.8
13	144.0		
14	41.6		
15	25.4		
16	27.8		
17	36.9		
18	44.5		
19	46.0		
20	30.1		
21	41.2		
22	74.0		
23	22.2		
24	62.3		
25	15.3		
26	16.5		
27	24.9		
28	20.3		
29	32.6		
30	28.2		
